# The Effect of Substrate Elasticity and Actomyosin Contractility on Different Forms of Endocytosis

**DOI:** 10.1371/journal.pone.0096548

**Published:** 2014-05-01

**Authors:** Dimitris Missirlis

**Affiliations:** Department of New Materials and Biosystems, Max Planck Institute for Intelligent Systems, Stuttgart, Germany, and Department of Biophysical Chemistry, University of Heidelberg, Heidelberg, Germany; University of California, San Diego, United States of America

## Abstract

Substrate mechanical properties have emerged as potent determinants of cell functions and fate. We here tested the hypothesis that different forms of endocytosis are regulated by the elasticity of the synthetic hydrogels cells are cultured on. Towards this objective, we quantified cell-associated fluorescence of the established endocytosis markers transferrin (Tf) and cholera toxin subunit B (CTb) using a flow-cytometry based protocol, and imaged marker internalization using microscopy techniques. Our results demonstrated that clathrin-mediated endocytosis of Tf following a 10-minute incubation with a fibroblast cell line was lower on the softer substrates studied (5 kPa) compared to those with elasticities of 40 and 85 kPa. This effect was cancelled after 1-hour incubation revealing that intracellular accumulation of Tf at this time point did not depend on substrate elasticity. Lipid-raft mediated endocytosis of CTb, on the other hand, was not affected by substrate elasticity in the studied range of time and substrate elasticity. The use of pharmacologic contractility inhibitors revealed inhibition of endocytosis for both Tf and CTb after a 10-minute incubation and a dissimilar effect after 1 hour depending on the inhibitor type. Further, the internalization of fluorescent NPs, used as model drug delivery systems, showed a dependence on substrate elasticity, while transfection efficiency was unaffected by it. Finally, an independence on substrate elasticity of Tf and CTb association with HeLa cells indicated that there are cell-type differences in this respect. Overall, our results suggest that clathrin-mediated but not lipid-raft mediated endocytosis is potentially influenced by substrate mechanics at the cellular level, while intracellular trafficking and accumulation show a more complex dependence. Our findings are discussed in the context of previous work on how substrate mechanics affect the fundamental process of endocytosis and highlight important considerations for future studies.

## Introduction

Cells respond to the mechanics of their microenvironment by transducing mechanical cues to biochemical information that contributes to the regulation of their adhesion, migration and differentiation [1], [2]. How this is mechanistically achieved is a subject of intense study, given the significance of mechanotransduction in development and disease progression [3], [4]. We here tested the hypothesis that cells regulate differentially endocytotic pathways in response to substrate elasticity as a potential way to modulate signals from the extracellular matrix.

Endocytosis is the process by which cells internalize extracellular material along with surrounding fluid by engulfing part of their plasma membrane. Endocytosis can be subdivided in several categories, according to the cell machinery and components necessary [5], [6]; among these, clathrin-mediated endocytosis is the most studied pathway, characterized by formation and internalization of clathrin-coated pits [7]. However, many molecules and viruses utilize clathrin-independent pathways to enter cells after binding to the plasma membrane [8].

Beyond providing cells with essential nutrients, endocytosis has numerous additional physiological functions. Cells exploit this process to regulate membrane receptor presentation, trafficking and control spatiotemporally signaling and thus sense their environment [6], [9]. Endocytosis further provides a mechanism to introduce specific biomolecules, such as drugs or genes, inside cells [10]. On the other hand, pathogens and viruses hijack the endocytic machinery to gain access in the cell interior and blocking this process is a promising intervention strategy [11]. The above highlight the paramount need to understand the internalization processes and the factors that control it.

The majority of our knowledge on endocytosis to date stems from *in vitro* studies performed on cells cultured on very rigid substrates, like plastic or glass. The focus has been on the influence of biochemical parameters while biomechanical factors originating from the substrate have been neglected, even though indications exist that they could be important in the process. In particular, studies have implicated several proteins that are involved in cell adhesion and that depend on cytoskeleton contractility, as endocytosis regulators [12], [13]. Moreover, physical parameters, such as membrane tension, are important determinants of endocytosis [14], [15].

Very few studies to date have addressed potential effects of substrate mechanics on endocytosis regulation *in vitro* [16], [17]. These studies suggested that internalization of different nanosized materials or drug delivery vehicles depended on the elasticity of the substrate cells were cultured on. The first report dates back to 2005, when Kong et al. investigated transfection efficiency of PEI-DNA complexes on preosteoblasts as a function of substrate elasticity [16]. Using fluorescence microscopy to quantify uptake, they claimed that internalization of the polymeric carrier increased with substrate elasticity in the range from 20 to 80 kPa. More recently, Huang *et al.* concluded that endothelial cells internalized carboxylated polystyrene nanoparticles (NPs) with a diameter of 100 nm at greater numbers when adhered on stiffer gels 6 kPa but the amount of NPs/ unit cell area decreased with stiffness [17].

Accordingly, we here set out to investigate whether distinct forms of endocytosis are regulated by the elasticity of the cell substrate. Beyond providing insight on cell mechanosensing, a possible correlation would provide a convenient means to regulate endocytosis and associated gene and drug delivery *in vitro* [18]. Additionally, it could have implications for the more complex *in vivo* situation; differences in tissue elasticity, especially between healthy and pathological sites, such as in injured arteries [19] or tumor tissue [20], could constitute a notable parameter when considering drug delivery to cells in such tissues.

Previous studies were conducted with engineered nanomaterials for which the internalization pathways were not specified. They did not therefore provide insight on the intriguing possibility that some forms of endocytosis are regulated by substrate elasticity, while others not. Moreover, the analysis was based on fluorescence microscopy imaging, which is limited in respect to the number of cells analyzed and prone to biased selection during acquisition. In addition, only 2-dimensional fluorescent images of cells were analyzed, neglecting cell volume effects.

In order to overcome the aforementioned shortcomings, we investigated the dependence of substrate elasticity on different forms of endocytosis by using established endocytosis markers and flow cytometry to analyze cell-associated fluorescence. This technique has the advantage of analyzing a much higher number of cells/experiment (we analyzed a minimum of 5000 cells; typically 10,000) in an unbiased fashion. Our results with specific endocytosis markers revealed that substrate elasticity in the range between 5 and 85 kPa affected initial (10 minute) clathrin mediated but not lipid-raft mediated internalization by a fibroblast cell line. At the same time, endocytosis marker accumulation over a longer incubation period of 1 hour did not show significant dependence on substrate elasticity. Interestingly, pharmacological manipulation of actomyosin contractility on cells cultured on plastic had a similar effect on both clathrin-mediated and lipid-raft mediated endocytosis after a 10-minute incubation time, but showed differential effects after 1-hour incubation, depending on the inhibitor used. The experimental protocol was next extended to different model compounds, including nanoparticles, which exhibited a elasticity-dependent cell association. Finally, experiments with HeLa cells demonstrated cell-type differences in respect to the impact of substrate elasticity on endocytosis.

## Results & Discussion

### Receptor- and lipid raft-mediated endocytosis do not depend on substrate elasticity

We utilized fibronectin (FN)-coated, PEG-based hydrogels as substrates with tunable elasticity as recently reported from our group [21]. Substrate elasticity is controlled by the polymer network architecture and density, independently of the amount of FN chemically immobilized on top of the gels [21]. Our experimental setup required seeding of up to 2×10^4^×5×10^4^ cells/gel; we therefore adapted our preparation protocol in order to form hydrogels on circular supports (PDMS or glass) ranging from 20 to 30 mm in diameter, corresponding to surface areas of 1.6 to 2.4 cm^2^, respectively. This way, we ensured sub-confluent conditions and minimized possible effects of mechanical influence coming from neighboring cells, directly or through the substrate [22]. Gels with three different values of elasticity (± standard deviation) were used: soft: 5.5 (±1.1), intermediate: 40.3 (±5.5) and stiff: 85.1 (±1.6) kPa (Figure S1). In addition, we here confirmed homogeneity of gels using AFM force spectroscopy to evaluate elasticity values on a 100 µm×100 µm gel area (Figure S1).

**Figure 1:**
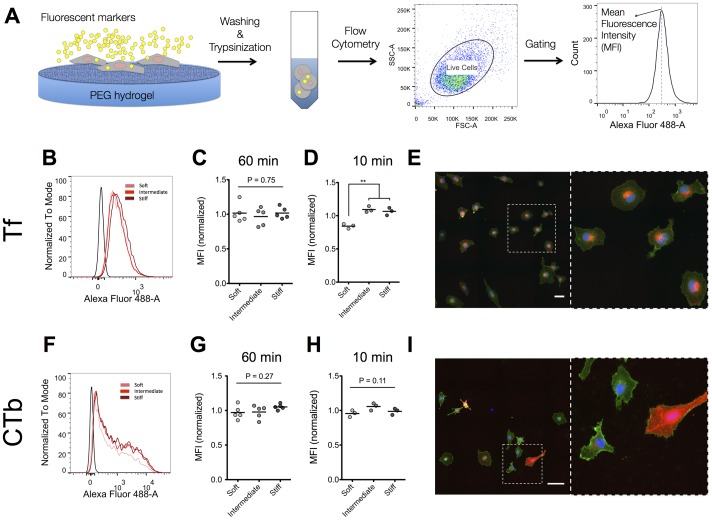
Substrate elasticity significantly affects association of Tf with REF52 cells after a 10-minute but not 1-hour incubation but does not have a significant effect on CTb association. (A) Schematic representation of experimental setup used to quantify cell association of fluorescent markers. Cells on PEG hydrogels of variable elasticity were incubated for a specified duration with fluorescent markers, washed to remove non-associated markers, detached using trypsin and analyzed using flow cytometry. Histograms of cell-associated fluorescence were acquired after gating live cells. (B) Histograms of a typical experiment showing efficient internalization of Tf by cells on hydrogels compared to negative controls and exhibiting a narrow Gaussian distribution. Tf association with REF52 cells was similar on soft, intermediate and stiff substrates after 1 hour incubation (C) but showed a significant lower normalized MFI on softer gels at the shorter time point of 10 minutes (D). Dot plots and mean values are presented, with each dot representing an independent experiment. (E) Epifluorescence microscopy images of REF52 cells on substrates with intermediate stiffness incubated 1 hour with Alexa Fluor 568-labeled Tf and stained for plasma membrane (WGA) confirmed that uptake of Tf by REF52 cells was uniform among the cell population and further showed that Tf localized in a perinuclear region of the cells. Scale bar: 100 µm (F) Histograms of a typical experiment of CTb association with REF52 cells showing a broad distribution of intensity value/cell. Normalized MFI values of CTb association were similar on soft, intermediate and stiff substrates after both 1 hour (G) or 10 minute (H) incubation with CTb. Dot plots and mean values are presented with each dot representing an independent experiment. (I) Epifluorescence microscopy images of REF52 cells on substrates with intermediate stiffness incubated 1 hour with Alexa Fluor 568-labeled CTb and stained for plasma membrane (WGA) showed a heterogeneous population of cells in respect to CTb internalization. Scale bar: 100 µm.

The REF52 fibroblast cell line was initially used to study the effect of substrate elasticity on different forms of endocytosis. We selected this cell line because we recently demonstrated that REF52 cells are responsive in the studied range of substrate elasticity in terms of adhesion and motility [21]. REF52 cells were cultured overnight on substrates of differing elasticity prior to uptake experiments of two different endocytosis markers with established binding receptors and well-characterized internalization pathways. Transferrin (Tf) and cholera toxin subunit B (CTb) conjugated to alexa fluor 488 (AF488) were incubated for 1 hour with cells and cell-associated fluorescence determined by flow cytometry analysis (Figure 1A).

Tf is a marker of receptor-mediated endocytosis that requires clathrin-coated vesicle formation and internalization [5]. Since the Tf receptor (TfR) is constitutively endocytosed, Tf is generally accepted as a marker for clathrin-mediated endocytosis [5], [23]. Association of Tf after 1 hour with REF52 cells did not significantly depend on substrate elasticity in the range studied (Figure 1B, C). The histograms obtained from flow cytometry analysis of Tf association exhibited a typical Gaussian shape, with coefficient of variation (CV) values of approximately 50%, indicating that Tf association was homogeneous among the cell population (Figure 1B). Immunofluorescence microscopy verified the homogeneous uptake of Tf by cells and qualitatively confirmed our flow cytometry results (Figures 1E and S2). Tf was mostly accumulated in intracellular vesicles at a perinuclear site.

Cholera toxin subunit B (CTb) binds ganglioside GM1, which is present in lipid rafts on the cell membrane and is internalized in a cholesterol-dependent manner by cells [24]. Again, there was no significant dependence of CTb association on substrate elasticity noted (Figure 1G). Flow cytometry histograms in this case revealed a broad peak characterized by large CV values of 150–250%, suggesting large variations between cells in respect to CTb association (Figure 1F). This heterogeneity was also clearly visible by immunofluorescence microscopy (Figures 1I and S3), highlighting the importance to analyze a large number of cells to obtain accurate quantification of average uptake.

In order to estimate the fraction of marker internalization, we quantified the amount of accessible Tf or CTb on the cell surface prior to flow cytometry analysis by using an antibody against AF488 that quenches its fluorescence. We first verified that the antibody quenched >90% of AF488 fluorescence at concentrations that largely exceeded the ones expected to be present on cells in our experiments (Figure S4). Addition of the quenching antibody in suspended cells after 1-hour incubation with Tf or CTb, and incubation for 15 min under ice revealed that approximately 10% of fluorescence was quenched in the case of Tf and 40% in the case of CTb association (Figure S4). The percentage of extracellular fluorescence did not depend on the elasticity of the substrate cells were cultured on, indicating similar binding and internalization kinetics, independent of substrate elasticity (Figure S4). Moreover, confocal microscopy z-stacks of cells incubated with Tf or CTb qualitatively confirmed the flow cytometry results, with fluorescence observed in the interior of cells (Figure S5). We can therefore conclude that most of the Tf associated with cells is intracellular (90%), while a higher fraction of CTb is adsorbed on the cell surface.

Our experimental data so far indicated that substrate elasticity in the examined range did not influence the amount of internalized Tf or CTb by REF52 cells after 1-hour incubation. Tf uptake and recycling occurs within a few minutes following its association with the TfR [25]; this raised the concern that after 1 hour, the amount of internalized Tf is not indicative of the kinetics of clathrin-mediated endocytosis, but instead expresses the accumulation of the Tf-TfR complex inside cells or a steady-state in Tf association. Similarly, CTb internalization occurs within minutes of association with the plasma membrane [26]. Therefore, we next studied Tf and CTb association following a shorter incubation time of 10 minutes. Interestingly, at this time point, association of Tf was significantly lower for cells cultured on the softer substrates (Figure 1D). Since the extracellular fraction of Tf did not differ for cells on the different substrates at this time point as well (Figure S4), our data indicated that endocytosis of Tf was dependent on substrate elasticity. In the case of CTb we did not observe any significant effect of substrate elasticity on its association with cells (Figure 1H) or its extracellular fraction (Figure S4) at the 10-minute point.

Overall, our results so far indicate that the kinetics of Tf internalization by REF52 cells is regulated by substrate elasticity in the examined range of elasticity values, but cellular association of Tf is equalized after 1-hour. On the other hand, lipid-raft mediated endocytosis does not appear to depend on substrate elasticity in REF52 cells under the experimental conditions studied.

### The effect of contractility inhibitors on uptake of Tf and CTb

**Figure 2:**
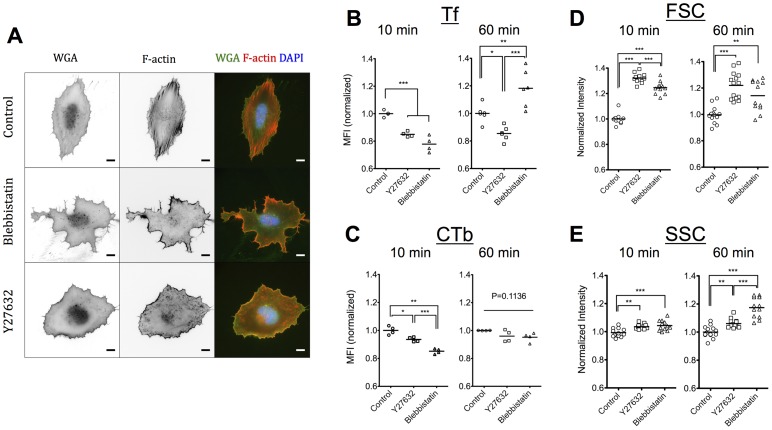
Inhibition of actomyosin contractility has a major effect on cell morphology and F-actin cytoskeleton but only a moderate, time-dependent influence on Tf and CTb association. (A) Epifluorescence microscopy images of REF52 cells 6 hours after seeding on FN-coated glass and stained for plasma membrane (WGA) and F-actin cytoskeleton (Phalloidin-TRITC). Cells were treated for 1 hour with 10 µM Y27632 or 50 µM blebbistatin, or left untreated as controls. Scale bars: 10 µm. Normalized MFI values for Tf (B) or CTb (C) association in presence or absence of 10 µΜ Y27632 or Blebbistatin, after a 10-minute or 1-hour incubation. Dot plots and mean values are presented. Normalized FSC (D) and SSC (E) intensity values of REF52 cells in presence or absence of 10 µΜ Y27632 or Blebbistatin as determined by flow cytometry analysis. Dot plots and mean values are presented with each dot representing an independent experiment.

Substrate elasticity regulates stress fiber formation and associated cell contractility, with cells on softer substrates being less contractile [27]–[31]. At the same time, a more drastic inhibition of contractility is possible through pharmacological treatment of cells cultured on rigid substrates [32]. Even though inhibition of contractility does not necessarily reproduce the phenotype of cells cultured on soft gels [33], we used this approach to investigate whether among the consequences of altered substrate elasticity, a pronounced reduction of actomyosin contractility affected different forms of endocytosis. Towards this end, we used two established contractility inhibitors: Y27632, a Rho-kinase specific inhibitor [34], which acts upstream of myosin II activity and blebbistatin, a non-muscle myosin II inhibitor [35]. First, we verified that incubation of REF52 cells with 10 µM Y27632 or 50 µM blebbistatin abrogated stress fiber formation. In accordance to previous studies, blebbistatin-treated and Y27632-treated fibroblasts no longer showed detectable stress fibers [32], [34] while blebbistatin-treated cells additionally exhibited an irregular morphology (Figure 2A) [36].

Association of Tf and CTb after a 10- or 60-minute incubation, in the presence or absence of inhibitors was quantified using flow cytometry as above. After 10 minutes, a significant decrease of Tf association was observed in both Y27632-treated cells (15%) and blebbistatin-treated cells (22%) (Figure 2B). Tf uptake inhibition in presence of Y27632 persisted after 1 hour, whereas an unexpected reversal in Tf association was observed in presence of blebbistatin (Figure 2B). The dependence of CTb association by contractility inhibitors was less pronounced (Figure 2C). An inhibition of 6% and 15% for cells treated with Y27632 or blebbistatin, respectively, was recorded at 10 minutes, with the effects attenuated after 1 hour (Figure 2C). These results demonstrated that actomyosin contractility regulates both clathrin-mediated and lipid-raft mediated endocytosis, with the effects being more pronounced in the former case. It is tempting to speculate that the dependence of Tf internalization observed on the softer PEG hydrogels at 10 minutes is due to reduced cell contractility; however, there are many additional factors beyond cell contractility that are regulated by substrate elasticity and further work is required to substantiate this hypothesis.

It is important to note that the distinct, time-dependent effect for the two inhibitors point towards discrete mechanisms of regulation of endocytosis and intracellular trafficking. Altered Tf recycling induced by blebbistatin could explain the higher accumulation of Tf within cells after 1 hour, since under control conditions TfRs are expected to recycle several times to the plasma membrane in this time frame [7], [25]. Indeed, when we monitored the kinetics of Tf association with REF52 cells cultured on plastic beyond 1 hour, in presence or absence of blebbistatin, we observed a reversal in blebbistatin effect from inhibition at shorter times (<30 minutes) to pronounced enhancement of Tf association after 2 hours (Figure S6). Moreover, the linear increase in Tf association in presence of blebbistatin during 2 hours, compared to the plateau reached under control conditions, further indicated deregulated recycling (Figure S6). Again, it remains to be confirmed whether this behavior in presence of myosin II inhibition could be linked to the loss of substrate elasticity dependence in Tf association after 1 hour (Figure 1C).

Previously, a few reports on the effect of the cell contractility inhibitors used in our study have been published. The uptake of a poly(ethelene imine)-DNA complexes by mMSCs after 2-hour incubation was inhibited by Y27632 to a small but significant extent (approximately 8%) [37]. Given that these complexes were primarily taken up by clathrin-mediated endocytosis [38], this results agrees well to the one recorded in our study for Tf uptake. On the other hand, a more pronounced decrease in the order of 50% was reported for Tf uptake by Y27632-treated T lymphocytes [39]. The authors claimed that Tf was still able to bind the Tf receptor but uptake was inhibited based on fluorescence studies [39]. We here tested this hypothesis by utilizing the anti-AF488 quenching antibody to compare the fraction of extracellular Tf on control cells *versus* cells treated with Y27632 or blebbistatin (Figure S4). There was no difference in the fraction of quenched fluorescence (approx. 10%) suggesting that cell-bound Tf is internalized at the same extent on treated cells. Moreover, confocal microscopy did not reveal any major differences in Tf localization in REF52 cells treated with Y27632 compared to controls (Figure S5). Therefore, the higher extent of uptake inhibition in the study of Samaniego et al. could be specific to the cell-type used and/or the experimental protocols and quantification methods used.

Studies investigating the effect of blebbistatin on different forms of endocytosis reported contradictory results. Samaniego et al. showed a decrease in Tf uptake (5 and 10 min incubation) by blebbistatin-treated T lymphocytes, similar to the one observed after Y27632 treatment as discussed above [39]. The similarity in the extent of Tf inhibition between the two inhibitors at these short time points agrees with our results. On the other hand, Holt et al. observed a small but significant increase in the association of a fluid-phase dextran marker, 30 minutes after incubation with dendritic cells [40]. The increase of intracellular marker compared to control conditions at this latter time point is in agreement with our hypothesis that blebbistatin affects intracellular trafficking processes following the initial endocytosis event.

### Flow cytometry reveals differences in scattering properties of cells treated with contractility inhibitors or cultured on soft substrates

Interestingly, the scattering properties of cells treated with contractility inhibitors as determined by flow cytometry were significantly different from those of untreated cells. Both signals from the forward scatter detector (FSC) and the side scatter detector (SSC) significantly increased for Y27632-and blebbistatin-treated REF52 cells (Figure 2D, E). The FSC is proportional to cell size, indicating that cells with inhibited contractility are larger, which was expected for detached cells in suspension, since actomyosin contractile forces pull on the plasma membrane and reduce the cell volume. The SSC signal depends on various parameters of cell morphology (e.g. granularity) and it is not possible at this point to attribute the effect of contractility inhibition on a particular parameter. Differences between the two inhibitors were also noted, further highlighting their differential effects on cell functions and morphology.

It is interesting to note that the value of SSC increased for cells cultured on the intermediate and stiff substrates compared to the soft ones (Figure S7). This result suggests that additional factors besides cell contractility affect SSC. Nevertheless, the significant dependence of REF52 SSC signal on substrate elasticity substantiates the fact that cells are responsive to this physical parameter in the studied range. The FSC values of REF52 cells remained unaltered in the elasticity range studied (Figure S7).

### Nanoparticle uptake 1-hour post-incubation depends on substrate elasticity

We next decided to extend our experimental procedure to study cell association of two different model compounds by REF52 cells. We studied cell association after 1-hour incubation of ***1*** *)* a high molecular weight (MW = 70,000) fluorescent dextran that does not have a specific extracellular receptor and therefore served as a non-specific pinocytosis marker and ***2*** *)* carboxylate-modified, fluorescent, latex nanoparticles (NPs) with a diameter of 100 nm that served as model drug delivery carriers.

**Figure 3:**
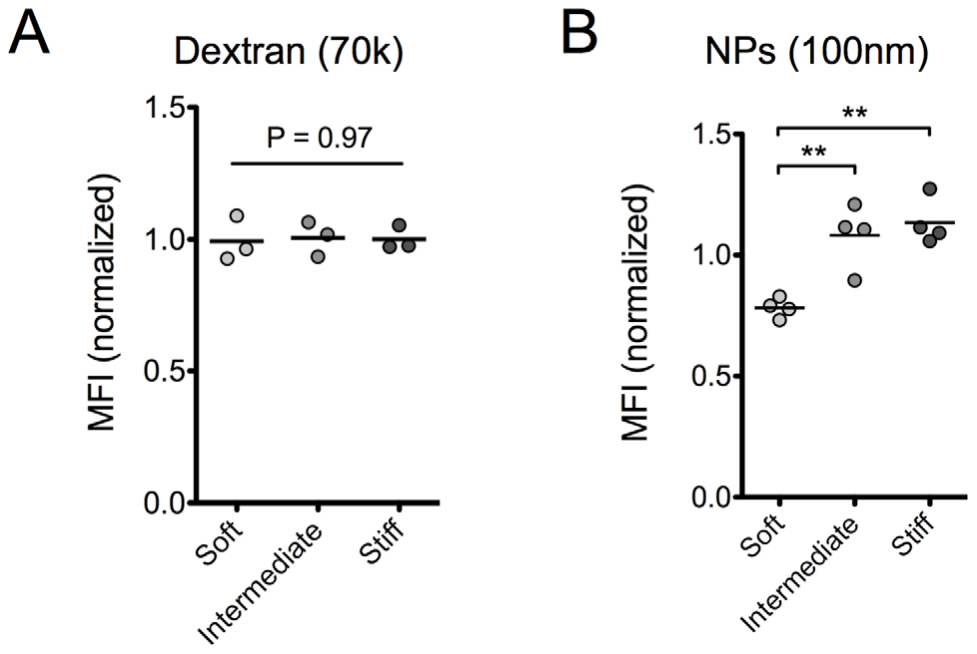
Association of latex nanoparticles but not of high MW dextran with REF52 cells depends on substrate elasticity. Normalized MFI values of REF52 cells incubated for 1(A) dextran (MW = 70,000) or (B) carboxylate-modified, polystyrene nanoparticles, 100 nm in diameter, as a function of substrate elasticity. Dot plots and mean values are presented with each dot representing an independent experiment.

The amount of associated dextran was low compared to association of specific markers and therefore a high concentration (250 µg/ml) of dextran was required for the uptake experiments. Flow cytometry analysis revealed a lack of dependence on substrate elasticity after 1-hour incubation, indicating that non-stimulated extracellular fluid uptake was not regulated by this parameter under our experimental conditions (Figure 3A).

On the other hand, uptake of NPs depended on substrate elasticity as indicated by the lower mean fluorescent intensity (MFI) of cells cultured on the softer gels (Figure 3B). This finding agrees with the trend observed by Huang et al. using the same type of nanoparticles, but substrates at a lower elasticity range [17]. Since this type of nanoparticles has been shown to be mainly internalized by clathrin-mediated endocytosis [41], [42], this finding agrees with our conclusion that clathrin-mediated endocytosis is regulated by substrate elasticity. However, we should note the difference in time of observation for the two materials, especially in view of the lack of dependence for Tf association at the 1-hour time point. This apparent discrepancy is most likely due to the different size of the internalized material, which has been shown to affect binding kinetics [43], uptake kinetics [44], [45] and the recycling of the internalized materials and/or receptors [44], [46]. Tf has a MW of approximately 80 kDa corresponding to a radius of gyration of around 3 nm [47], much smaller than that of the 100 nm NPs. In the future, it would be interesting to compare internalization of Tf and Tf-conjugated NPs of different sizes in order to better understand the combined effects of size and substrate elasticity on clathrin-mediated endocytosis.

### Transfection efficiency of cells was not influenced by substrate elasticity

Next, we asked whether transfection efficiency using a commercially available transfection agent (Promofectin) was dependent on substrate elasticity. Previously, it was suggested that transfection efficiency is regulated by substrate elasticity [16] and since the first step in transfection is the internalization of the plasmid DNA-transfection agent complex, we were here interested in examining the dependence on substrate elasticity using our experimental setup. We transfected REF52 cultured on soft, intermediate or stiff PEG hydrogels with a GFP-expressing plasmid and determined the percentage of transfected cells 24 hours after plasmid addition. There were no significant differences observed in the fraction of transfected cells (Figure S8). This observation is at odds with previous studies [16]; this could be due to a large number of factors including the type of gene delivery vehicle, quantification method for transfection efficiency, cell type and culture conditions. It is important to note however, that our results demonstrate that non-viral gene delivery is not necessarily regulated by substrate elasticity and care should be taken when conclusions are generalized based on the results from a limited range of conditions.

### Endocytosis in HeLa cells is not dependent on substrate elasticity

**Figure 4:**
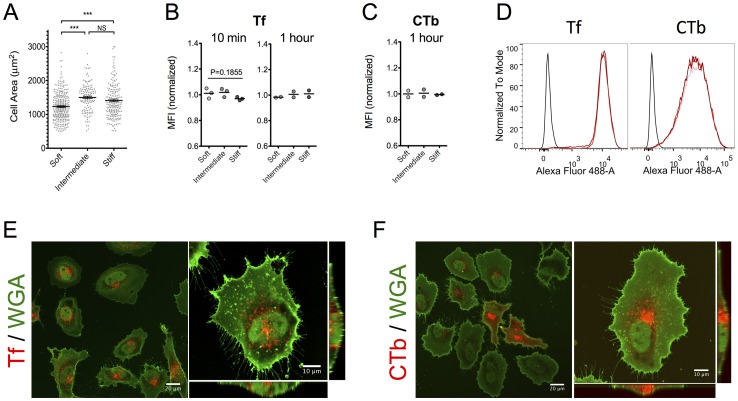
Association of Tf and CTb with HeLa cells is independent of substrate elasticity. (A) Projected cell area of HeLa cells spread on FN-coated hydrogels increased with substrate stiffness. More than 100 cells/condition were analyzed. (B) Normalized MFI values of HeLa cells incubated for 10 minutes or 1 hour with fluorescently labeled Tf indicated that its association with cells is not dependent on substrate elasticity. Dot plots and mean values are shown; each experiment is represented by a dot (n>10,000 analyzed cells/experiment). (C) Normalized MFI values of HeLa cells incubated for 1 hour with fluorescently labeled CTb indicated a lack of regulation of its association by substrate elasticity as well. (D) Histograms from a typical experiment showed that, similarly to REF52 cells, Tf association was more homogeneous compared to CTb association. (E, F) Confocal microscopy images of HeLa cells on FN-coated glass confirming homogeneous uptake in the case of Tf (E) and a broader distribution of fluorescence per cell for CTb (F). For both markers, the majority of fluorescence is intracellular. Scale bars 10 µm.

Finally, we asked whether our results on the association of the specific markers Tf and CTb as a function of substrate elasticity on endocytosis were valid for other cell types. HeLa is a human cancer cell line, widely used in pharmacological and drug delivery studies, and was used here for this purpose [48]. First, we examined HeLa adhesion and mechanosensing on FN-coated gels. Cells adhered efficiently on gels of differing elasticity (Figure S9) and showed significantly higher spreading on intermediate and stiff substrates compared to the softer substrates tested, indicating that HeLa cells respond to substrate mechanics in the elasticity range studied (Figure 4A). Flow cytometry revealed that association of Tf after a 10-minute or 1-hour incubation, or CTb after an 1-hour incubation was unaffected by substrate elasticity for this cell line (Figure 4B, C). HeLa cells internalized Tf and CTb more efficiently compared to REF52 cells as denoted by the approximately 100-fold increase in MFI in respect to non-treated cells for both markers in HeLa cells (Figure 4D) compared to a 10- to 20-fold increase for Tf and a 40-fold increase for CTb in REF52 cells (Figure 1B). Similar to fibroblasts, flow cytometry histograms indicated homogeneous association of Tf, whereas intensity vales for AF488-CTb per cell showed a broad distribution (Figure 4D). These observations were further confirmed by confocal microscopy images of HeLa cells cultured on FN-coated glass and incubated with Tf or CTb (Figure 4E, F). Finally, transfection efficiency for this cell line was also not regulated by substrate elasticity (Figure S8). The lack of correlation between substrate elasticity and internalization of the two specific markers for this cell line suggests that our findings are cell type specific. It is possible that the reason for this independence lies in the cancerous origin of HeLa cells; both deregulated endocytosis [49] and insensitivity to substrate mechanics for transformed cells [50] have been reported. Overall, our experimental setup should be extended to more cell types in the future, including primary cells, in order to clearly establish generalized dependencies of endocytosis on substrate elasticity.

### Considerations in the context of substrate elasticity dependence on endocytosis

**Figure 5:**
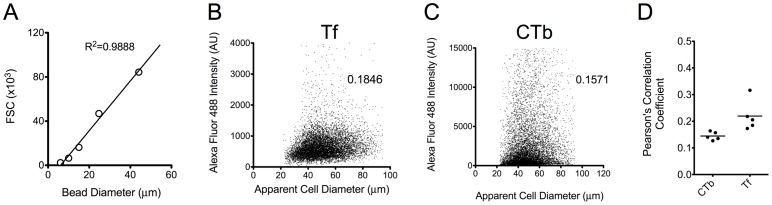
Association of Tf and CTb with REF52 cells is not correlated with cell size. (A) Different sized polystyrene beads were used to derive a linear relationship between bead diameter and the FSC detector signal. (B, C) Typical dot plots and correlation coefficients (Pearson's) for association of Tf (B) or CTb (C) and apparent cell diameter of live cells. (D) Results of Pearson's correlation coefficients for 5 different flow cytometry analyses are presented as dot plots and mean values. The low values (<0.3) indicate very low to no correlation between cell size and amounts of Tf or CTb associated with cells.

Our experimental methodology and results highlighted several important considerations in respect to the effects of substrate elasticity on different forms of endocytosis. ***First***, the analysis of a large number of cells using flow cytometry revealed major differences in homogeneity of uptake depending on the marker type. Consequently, it is important to analyze many cells in order to avoid artifacts associated with small sample sizes. ***Second***, in order to distinguish between internalization and accumulation of a particular endocytosis marker, which can depend on additional processes such as intracellular trafficking and exocytosis, early time points for analysis should be selected. ***Third*** *,* the use of quenching antibodies provided in a reproducible manner an estimation of the surface-exposed fluorescent markers, and therefore the internalized fraction. ***Fourth***, flow cytometry allowed us to examine the correlation between cell-associated fluorescence and cell size, as expressed by the FSC signal. We observed a weak correlation between these two parameters for both Tf and CTb association (Figure 5), independent of substrate elasticity. In order to relate FSC signal to cell size, we used calibration beads with diameters ranging from 6 to 44 µm and obtained a linear relationship between size and FSC (Figure 5A). Accordingly, we calculated the Pearson's correlation coefficient for the relationship between associated fluorescence of Tf or CTb and cell size from 5 random independent experiments (Figures 5B–D and S10). The low values of correlation coefficient (<0.3) indicated very weak to no correlation between these parameters. Overall, we concluded that marker internalization was largely independent on cell size and therefore care should be taken when using cell spread area to normalize uptake (for example in figure 5 in reference 17).

It is also important to note that our conclusions are valid for the particular conditions and cell lines used and their generality should be extended in the future towards other cell lines and setups. Our results were obtained using a PEG-based, FN-functionalized substrate, which has a different background chemical composition compared to materials employed in previous studies investigating the effect of substrate elasticity on internalization [16], [17]. Even though we have previously shown that fibroblasts respond to substrate elasticity on the gels used here in respect to their adhesion and migration behavior [21], we cannot exclude the possibility that differences in cell adhesive coating or underlying polymer structure are responsible for the presence or lack of correlation between marker uptake and substrate elasticity. Indeed, a dependence of ECM type on internalization and transfection efficiency has been reported [51] and the extent of cell responses on substrate elasticity greatly depends on the substrate material [52] and the type of cell-adhesive coating [21], [30], [53]–[55]. Future studies should expand the validity of our results on the stiffness dependencies in endocytosis to cells exposed to varying insoluble biochemical signals.

## Conclusions

In summary, we have shown that fibroblasts internalize Tf (a marker of clathrin-mediated endoyctosis) more efficiently on stiffer FN-coated hydrogels (40 and 85 kPa) compared to softer ones (5 kPa). However, intracellular accumulation of Tf after longer exposure (1 hour) did not depend on substrate elasticity. Accordingly, internalization of fluorescent NPs, previously shown to be internalized through the clathrin-mediated pathway, showed a similar dependence on substrate elasticity. On the other hand, association of CTb or high MW dextran molecules was unaffected by substrate elasticity in the examined range, indicating that different forms of endocytosis are not regulated in the same manner by the substrate. Pharmacological inhibition of actomyosin contractility had significant effects on uptake of Tf, depending on inhibitor type; treatment with blebbistatin initially inhibited its uptake, while at longer time points an accumulation of Tf inside blebbistatin-treated cells was observed compared to control cells. This finding suggested that myosin II activity affects both internalization and recycling kinetics and it remains to be seen whether the phenotype recorded on the softer gels is linked to a decrease in myosin II activity. Finally, the experimental data obtained with HeLa cells showed independence of Tf association on substrate elasticity, indicating that cell type specific differences exist in this respect. Overall, the results presented here suggest that substrate elasticity can potentially influence distinct endocytosis pathways and cargo internalization and that the approach we have introduced is suitable for future studies on the endocytosis of more specialized drug delivery systems or specific receptors as a function of substrate mechanics.

## Materials & Methods

### Hydrogel preparation & Characterization

PEG-based hydrogels were prepared via Michael-type addition, cross-linking reaction of 4-arm PEG precursors functionalized with vinyl sulfone groups (PEGVS) or thiol groups (PEGSH) as previously described [21], [56]. Briefly, PEGVS was mixed with PEGSH at a molar VS:SH ratio of 1:1.05, vortexed for 5 seconds and cysteamine (final concentration of 1 mM) was added to the mixture. The precursor solution (50 µl) was pipetted on a circular glass cover slip (30 mm in diameter) silanized with (3-aminopropyl)triethoxysilane (APTES; Sigma) or a custom-made PDMS well (24 mm in diameter), and a hydrophobic glass slide (treated with Sigmacote®, Sigma) was placed on top, separated by either Teflon spacers or aluminium foil. After 2 hours at room temperature, hydrogels were swollen in water and the hydrophobic glass removed carefully using a razor blade.

### Cell Culture

A rat fibroblast cell line (REF52) [21] and HeLa (human cervical cancer) cells [57] (kindly provided by Dr L. Roth, DKFZ, Heidelberg, Germany) were cultured as sub-confluent monolayers in Dulbecco's modified eagle's medium (DMEM), supplemented with 10% fetal bovine serum and 1% penicillin/streptomycin, at 37°C and 5% CO_2_ in a humidified atmosphere. REF52 cells were serum-starved overnight (12–16 hours) prior to seeding on FN-coated gels. For cell seeding, cells were detached with accutase for 5 minutes, centrifuged and the cell pellet was resuspended in non-supplemented DMEM at the desired cell concentration. After 30 minutes of incubation on top of substrates, non-adhered cells were removed by aspiration and supplemented DMEM added. HeLa cells were seeded in the same manner but were incubated in serum-free medium only 1 hour prior to seeding.

### Flow cytometry

The amount of associated fluorescence per cell was quantified using flow cytometry. Cells seeded on FN-coated gels or TCPS were washed once and incubated in DMEM without serum 30 minutes before each experiment. For measuring association of specific markers, cells were incubated for 10 minutes or 1 hour in non-supplemented DMEM with *1)* 10 µg/ml transferrin (Tf) from human serum, alexa fluor 488 conjugate (Life Technologies) or *2)* 1 ng/ml recombinant cholera toxin subunit B, alexa fluor 488 conjugate (Life Technologies). For non-specific pinocytosis cells were incubated for 1 hour with 250 µg/ml dextran (70,000 molecular weight), Oregon Green 488 conjugate (Life Technologies). Nanoparticle association studies were performed using commercially-available, fluorescent, carboxylate-modified poly(styrene) nanoparticles (NPs) (Fluospheres; Life Technologies) of 100 nm in diameter. Before addition to cells, NPs were sonicated for 5 minutes and diluted 1∶1000 to a concentration of 0.02 mg/ml. Cells were incubated for 1 hour with the NPs. For inhibition studies, the following inhibitors were added 30 minutes prior to marker addition: 1) 10 µM Y27632 (Sigma), 2) 50 µM blebbistatin (Sigma).

After incubation with markers or NPs, cells were washed three times with ice-cold PBS and detached from gels using trypsin-EDTA (5-minute incubation at 37°C). Cells were centrifuged (1500 rpm, 4 minutes) and the cell pellet resuspended in 0.25 ml PBS; cell suspension was kept under ice until flow cytometry analysis (20–60 min post-trypsinization). For quenching of extracellular fluorescence, an anti-Alexa Fluor 488 quenching antibody (10 µg/ml; Life Technologies) was added to the cell suspension for at least 15 min prior to flow cytometry analysis.

Flow cytometry was performed on a BD FACSCanto flow cytometer. A minimum of 5,000 events in a gate of live cells was recorded per sample (typically 10,000). Due to variations in absolute values between different experiments we normalized values as following: first, the mean fluorescent intenstity (MFI) of non-treated cells was subtracted from the MFI of the sample; then the average MFI value of all samples for a given experiment was calculated and the value of each sample was normalized to this average value. Polystyrene beads of different sizes (Polysciences Inc.) were used to correlate the forward scatter signal (FSC) to particle diameter.

### Microscopy

Phase contrast and epifluorescence imaging were performed using a Delta Vision system (Applied Precision Inc.) on an Olympus IX inverted microscope equipped with a cooled CCD camera. For high magnification images a 60×/1.3 NA (Olympus) oil-immersion objective was used. Epifluorescence microscopy was additionally performed using a Leica DM6000B upright microscope equipped with a CCD camera. A water immersion objective 40×/0.8 NA (Leica) was used. Laser scanning confocal microscopy (LCSM) was performed on a Zeiss LSM 5 microscope using a 40×/1.2 NA (Zeiss) water immersion objective.

Cells were fixed with 4% paraformaldehyde in PBS (15 minutes in PBS) and stained with fluorescent agents. Wheat germ agglutinin, AlexaFluo 488 conjugate (WGA; 10 µg/ml) was used to stain plasma membrane, phalloidin-tetramethyl rhodamine B isothiocyanate (Phalloidin-TRITC; 2.5 µg/ml) stained filamentous actin and 4′,6-diamidino-2-phenylindole (DAPI; 1.0 µg/ml) labeled cell nuclei. Projected cell area was calculated using the *Cell Outliner* plugin of ImageJ software (NIH) from fluorescence microscopy images of WGA-stained cells.

### Transfection experiment

Cells were cultured on top of FN-coated gels or glass for 24 h prior to transfection. A plasmid for transient transgene expression of green fluorescent protein (GFP) was purchased from Promokine [pPK-CMV-R4 Receptor Vector (GFP)]. Promofectin (PromoKine) was used to transfect cells according to manufacturers' protocol. Briefly, the plasmid (1∶20 in DMEM) was mixed with promofectin (1∶10 in DMEM) and incubated for 15 min at room temperature. Next the transfection complex was added (1∶70) to a glass-bottom dish containing multiple gels and a control glass surface in supplemented DMEM. In this way, the amount of available DNA/cell was the same for the different conditions in each experiment. The percentage of transfected cells was analyzed by immunofluorescence microscopy by dividing the number of GFP-positive cells to the total number of cells. Results of transfection efficiency on gels are normalized in respect to that on glass, since absolute values varied considerably between experiments.

### Statistical analyses

All statistical analyses were performed using the software Prism (GraphPad Inc.). Experiments of cell association on hydrogels of differing elasticity were statistically analyzed using the Tukey test, which compares all pairs of columns. Comparisons between control and samples treated with contractility inhibitors or quenching antibody were analyzed using an unpaired t-test. Only statistical significant differences are presented in graphs with p values <0.01, <0.05 and <0.001 represented as *, ** and ***, respectively.

## Supporting Information

Figure S1 **Mechanical characterization of PEG hydrogels using AFM.** (A) Young's moduli of three different gel formulations determined by AFM force spectroscopy using a spherical glass tip (diameter 10 µm) and the Hertz model to analyze the force-distance curves. The average values of 3 independent experiments (typically 2 gels per experiment and 3 positions on each gel analyzed) and the standard deviation are presented. (B) A surface area of 100 µm ×100 µm on gels was probed to evaluate homogeneity of elasticity. 100 points were analyzed (orthogonal grid) and 5 force curves/point were analyzed to yield the Young's modulus of that point. Data are presented as color-coded surface plots and reveal variations 5–10% among values.
(PDF)

Figure S2 **Internalization of Tf by REF52 cells is uniform and qualitatively similar between gels of differing elasticity.** Epifluorescence microscopy images (multiple stitched fields) of REF52 cells on PEG hydrogels of varying stiffness, incubated for 1 h with Alexa Fluor 568-conjugated Tf and plasma membrane stained with WGA. Homogeneous uptake of Tf by REF52 cells and intracellular localization was noted for all values of elasticity investigated. Scale bars: 50 µm.
(TIFF)

Figure S3 **Internalization of CTb by REF52 cells is heterogeneous among the cell population.** Epifluorescence microscopy images (multiple stitched fields) of REF52 cells on PEG hydrogels of varying stiffness, incubated for 1 h with Alexa Fluor 568-conjugated CTb and plasma membrane stained with WGA. The extent of CTb association with REF52 cells varied considerably between cells. However, the pattern of association was similar between hydrogels of differing elasticity for all values of elasticity investigated. Scale bars: 100 µm.
(TIFF)

Figure S4 **Estimation of extracellular marker fraction using anti-alexa fluor 488 (anti-AF488) quenching antibody.** (A) Quenching kinetics and efficiency of the anti-AF488 antibody (10 µg/ml) on a 5 nM (0.4 µg/ml) solution of AF488-Tf showed a maximal 90% quenching of fluorescence within 10 minutes of mixing. The antibody concentration used is the same as that used in cell experiments while AF488-Tf concentration is much higher compared to that on cell-associated Tf or CTb, as estimated by fluorescence measurements. (B) MFI of REF52 cells treated with anti-AF488 normalized to MFI in its absence. Incubation of REF52 cells with the quenching antibody on cells cultured on FN-coated TCPS revealed a 11% decrease in Tf MFI and 38% decrease in CTb MFI, indicating that approximately 90% of Tf and 60% of CTb are internalized (mean and standard deviations of at least 3 samples and 2 independent experiments). Substrate elasticity did not affect the fraction of internalized markers (n = 1). (C) The effect of Y27632 and blebbistatin treatment on the extracellular fraction of Tf was evaluated using anti-AF488. The same fraction of extracellular Tf was recorded independent of cell treatment. Mean and standard deviations are shown of 3 samples.
(TIFF)

Figure S5 **Rho kinase inhibition with Y27632 did not alter intracellular fluorescence pattern of Tf or CTb on REF52 cells.** Confocal microscopy images of REF52 cells on FN-coated glass after 1-hour incubation with AF568-labeled markers, fixation and staining with WGA-AF488. Tf was internalized at similar numbers by cells and mainly localized at a perinuclear site, independently of Y27632 treatment (upper row), while CTb showed heterogeneous uptake efficiency among the cell population that was also independent of Y27632 treatment (lower row).
(TIFF)

Figure S6 **Blebbistatin treatment of REF52 cells affects Tf internalization and recycling kinetics.** MFI of REF52 cells incubated with Tf at different time points on FN-coated plastic in the presence or absence of 50 µM blebbistatin. At short incubations blebbistatin inhibits Tf association by cells, while at longer time points the amount of Tf is enhanced compared to control conditions. The quasi-linear increase of MFI per cell indicates that blebbistatin has an effect of intracellular trafficking and recycling of Tf. Each data point represents the average of two experiments.
(TIFF)

Figure S7 **The SSC signal but not the FSC signal of REF52 cells depends on the elasticity of the substrate they were cultured on.** Flow cytometry analysis of REF52 cells cultured on gels did not show a dependence of their FSC signal (A), while cells on soft gels showed a significantly lower SSC signal compared to cells cultured on intermediate or stiff hydrogels (B). Values from at least 4 independent experiments are presented with the value from each gel represented by a single dot and the mean value a solid line.
(TIFF)

Figure S8 **Transfection efficiency of HeLa or REF52 cells is not significantly affected by substrate elasticity.** (A) Typical overlaid optical and fluorescence microscopy images of HeLa cells on FN-coated hydrogels of different elasticity used to calculate transfection efficiency. Insets show micrographs of the fluorescence channel (GFP). Scale bars: 50 µm. Transfection efficiency of (B) HeLa or (C) REF52 cells normalized to the value obtained on FN-coated glass. A non-significant decrease of the fraction of transfected cells with increasing stiffness was noted. Mean and SEM values from at least 3 gels and two independent experiments are presented (n = 200–1000 analyzed cells/gel).
(TIFF)

Figure S9 **Cell density of HeLa cells does not depend on substrate elasticity.** Inverted fluorescence microscopy images (stitched tiles) of HeLa cells on soft, intermediate and stiff hydrogels, stained with WGA. The amount of cells adhered was similar on all hydrogels, independent of their elasticity. Scale bars: 200 µm.
(TIFF)

Figure S10 **Tf or CTb association with cells is very weakly correlated to cell size.** Dot plots of cell-associated fluorescence as a function of apparent cell diameter for Tf (left) or CTb (right) association. Data from 5 random experiments performed on cells on top of gels or glass are presented, along with the calculated Pearson's correlation coefficient.
(TIFF)
