# Supplementary figures and images for: The Effect of Substrate Elasticity and Actomyosin Contractility on Different Forms of Endocytosis
Source: PLoS One. 2014 May 1;9(5):e96548. doi: 10.1371/journal.pone.0096548 (PMC4006897; doi:10.1371/journal.pone.0096548)

A

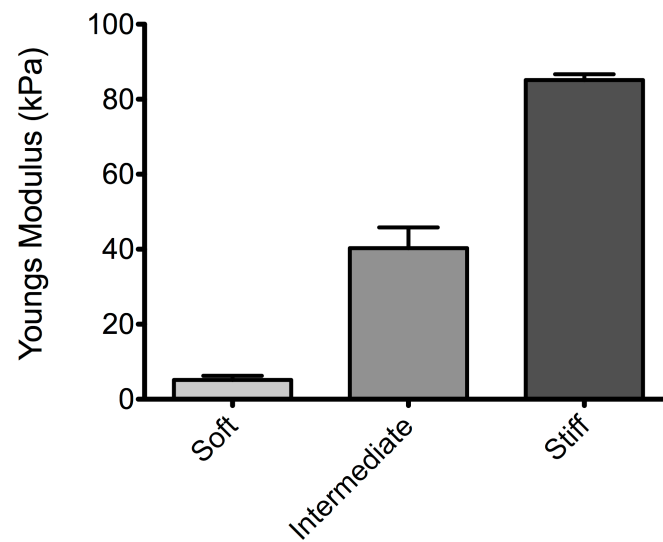

B

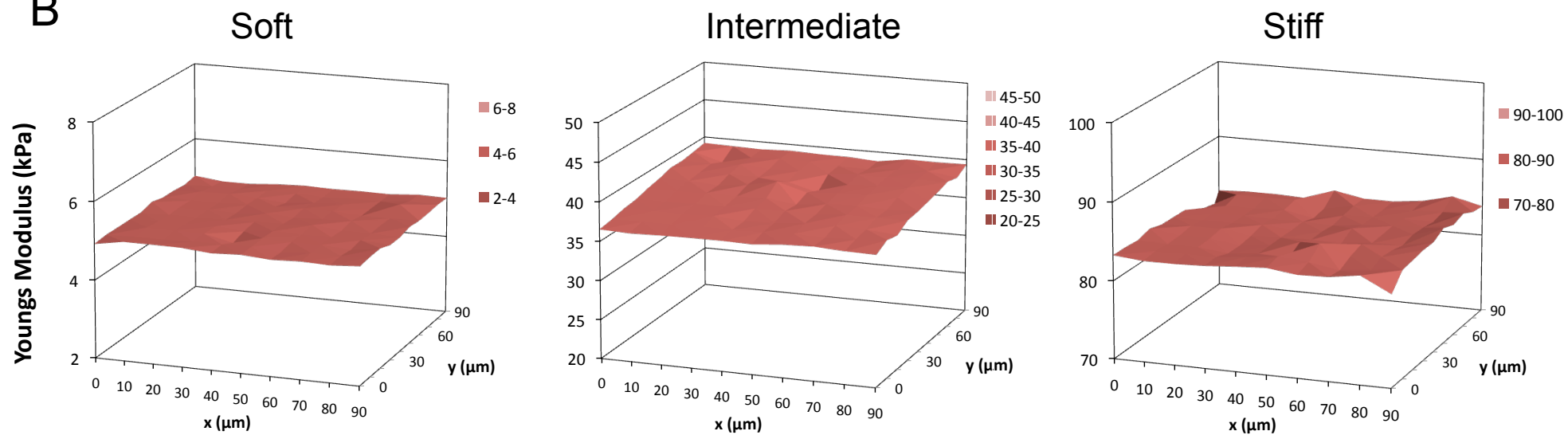

Supplement: Figure S1 — Mechanical characterization of PEG hydrogels using AFM. (A) Young's moduli of three different gel formulations determined by AFM force spectroscopy using a spherical glass tip (diameter 10 µm) and the Hertz model to analyze the force-distance curves. The average values of 3 independent experiments (typically 2 gels per experiment and 3 positions on each gel analyzed) and the standard deviation are presented. (B) A surface area of 100 µm ×100 µm on gels was probed to evaluate homogeneity of elasticity. 100 points were analyzed (orthogonal grid) and 5 force curves/point were analyzed to yield the Young's modulus of that point. Data are presented as color-coded surface plots and reveal variations 5–10% among values. (PDF) [file pone.0096548.s001.pdf]

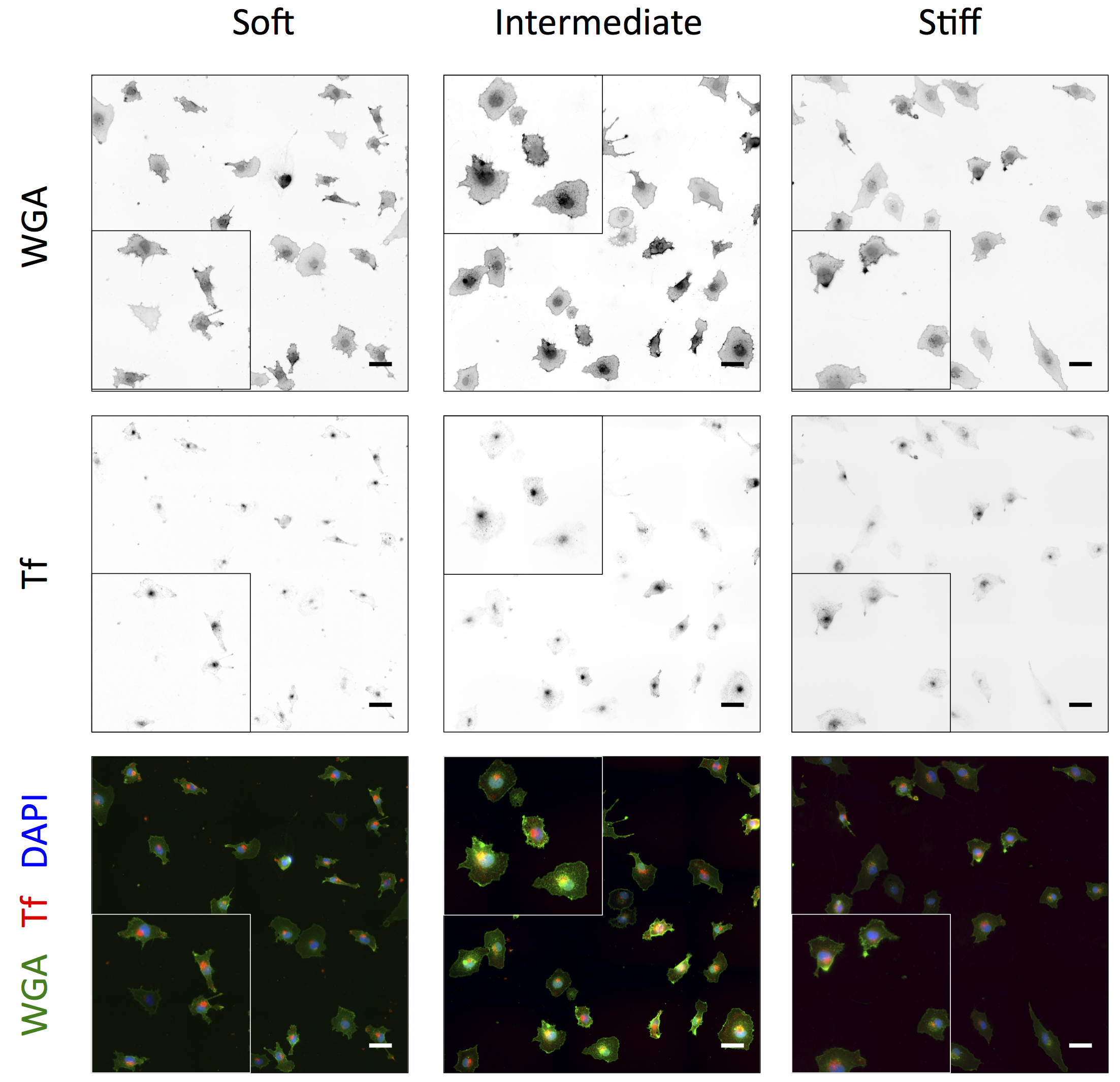

Supplement: Figure S2 — Internalization of Tf by REF52 cells is uniform and qualitatively similar between gels of differing elasticity. Epifluorescence microscopy images (multiple stitched fields) of REF52 cells on PEG hydrogels of varying stiffness, incubated for 1 h with Alexa Fluor 568-conjugated Tf and plasma membrane stained with WGA. Homogeneous uptake of Tf by REF52 cells and intracellular localization was noted for all values of elasticity investigated. Scale bars: 50 µm. (TIFF) [file pone.0096548.s002.tif]

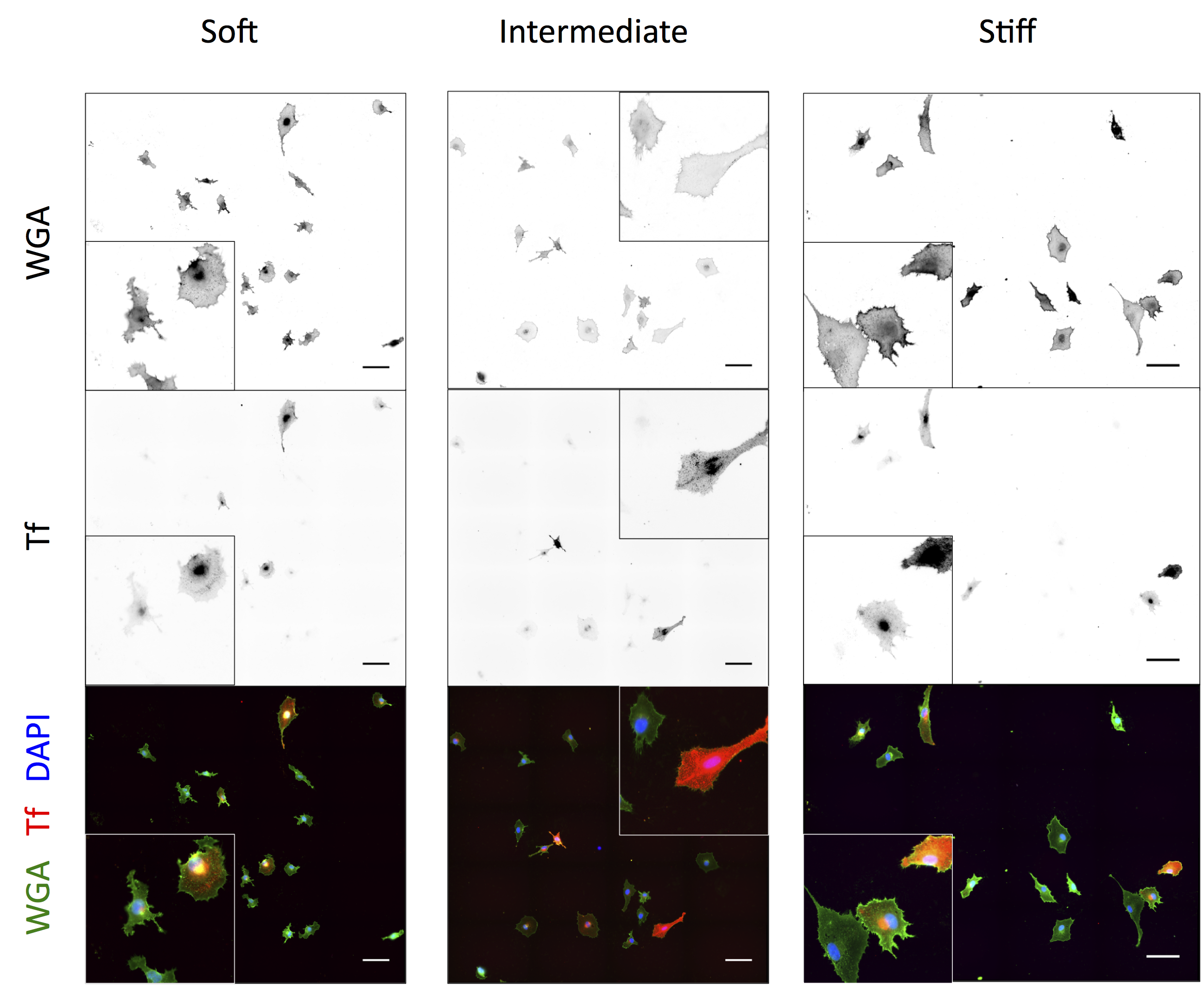

Supplement: Figure S3 — Internalization of CTb by REF52 cells is heterogeneous among the cell population. Epifluorescence microscopy images (multiple stitched fields) of REF52 cells on PEG hydrogels of varying stiffness, incubated for 1 h with Alexa Fluor 568-conjugated CTb and plasma membrane stained with WGA. The extent of CTb association with REF52 cells varied considerably between cells. However, the pattern of association was similar between hydrogels of differing elasticity for all values of elasticity investigated. Scale bars: 100 µm. (TIFF) [file pone.0096548.s003.tif]

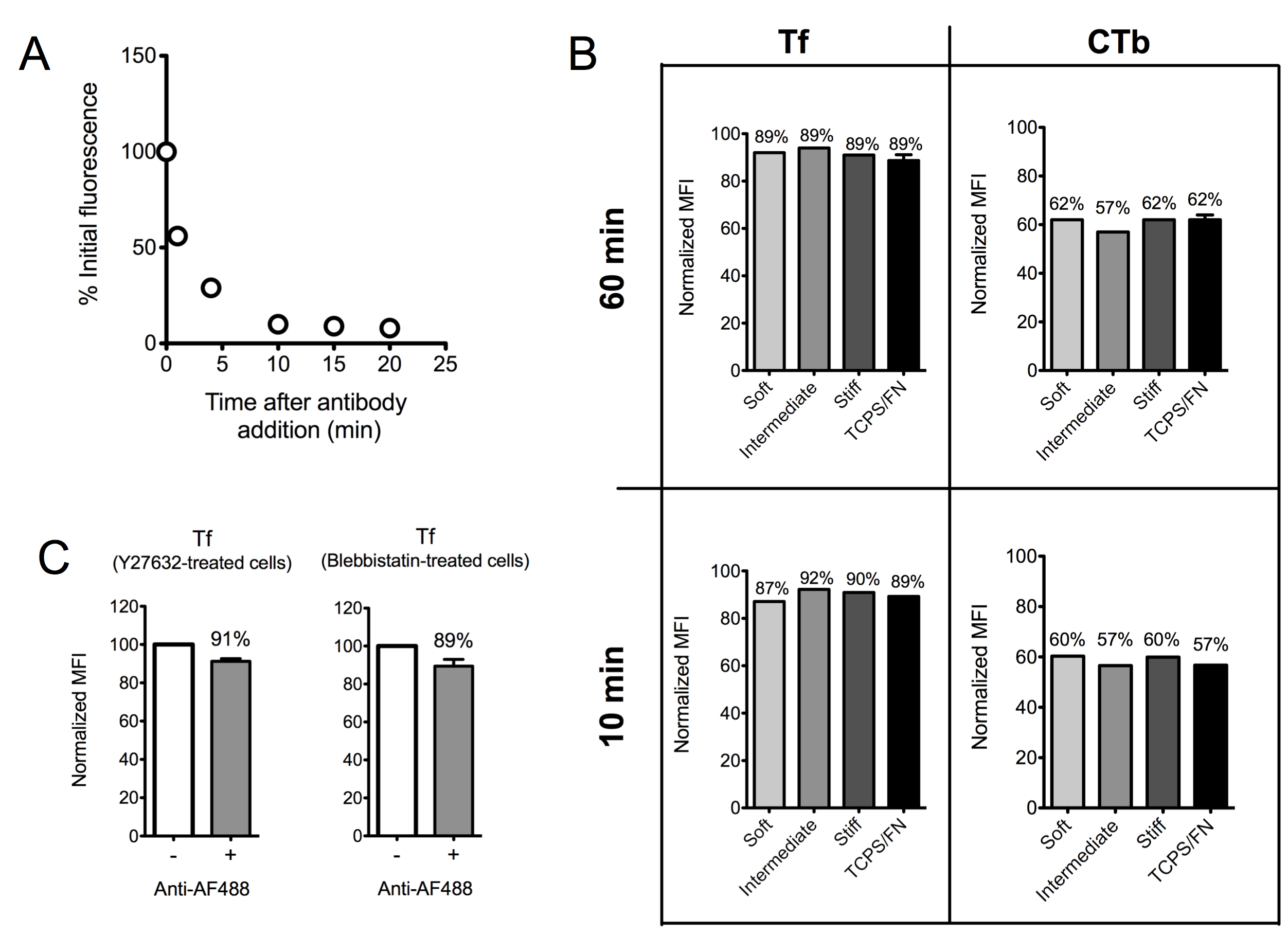

Supplement: Figure S4 — Estimation of extracellular marker fraction using anti-alexa fluor 488 (anti-AF488) quenching antibody. (A) Quenching kinetics and efficiency of the anti-AF488 antibody (10 µg/ml) on a 5 nM (0.4 µg/ml) solution of AF488-Tf showed a maximal 90% quenching of fluorescence within 10 minutes of mixing. The antibody concentration used is the same as that used in cell experiments while AF488-Tf concentration is much higher compared to that on cell-associated Tf or CTb, as estimated by fluorescence measurements. (B) MFI of REF52 cells treated with anti-AF488 normalized to MFI in its absence. Incubation of REF52 cells with the quenching antibody on cells cultured on FN-coated TCPS revealed a 11% decrease in Tf MFI and 38% decrease in CTb MFI, indicating that approximately 90% of Tf and 60% of CTb are internalized (mean and standard deviations of at least 3 samples and 2 independent experiments). Substrate elasticity did not affect the fraction of internalized markers (n = 1). (C) The effect of Y27632 and blebbistatin treatment on the extracellular fraction of Tf was evaluated using anti-AF488. The same fraction of extracellular Tf was recorded independent of cell treatment. Mean and standard deviations are shown of 3 samples. (TIFF) [file pone.0096548.s004.tif]

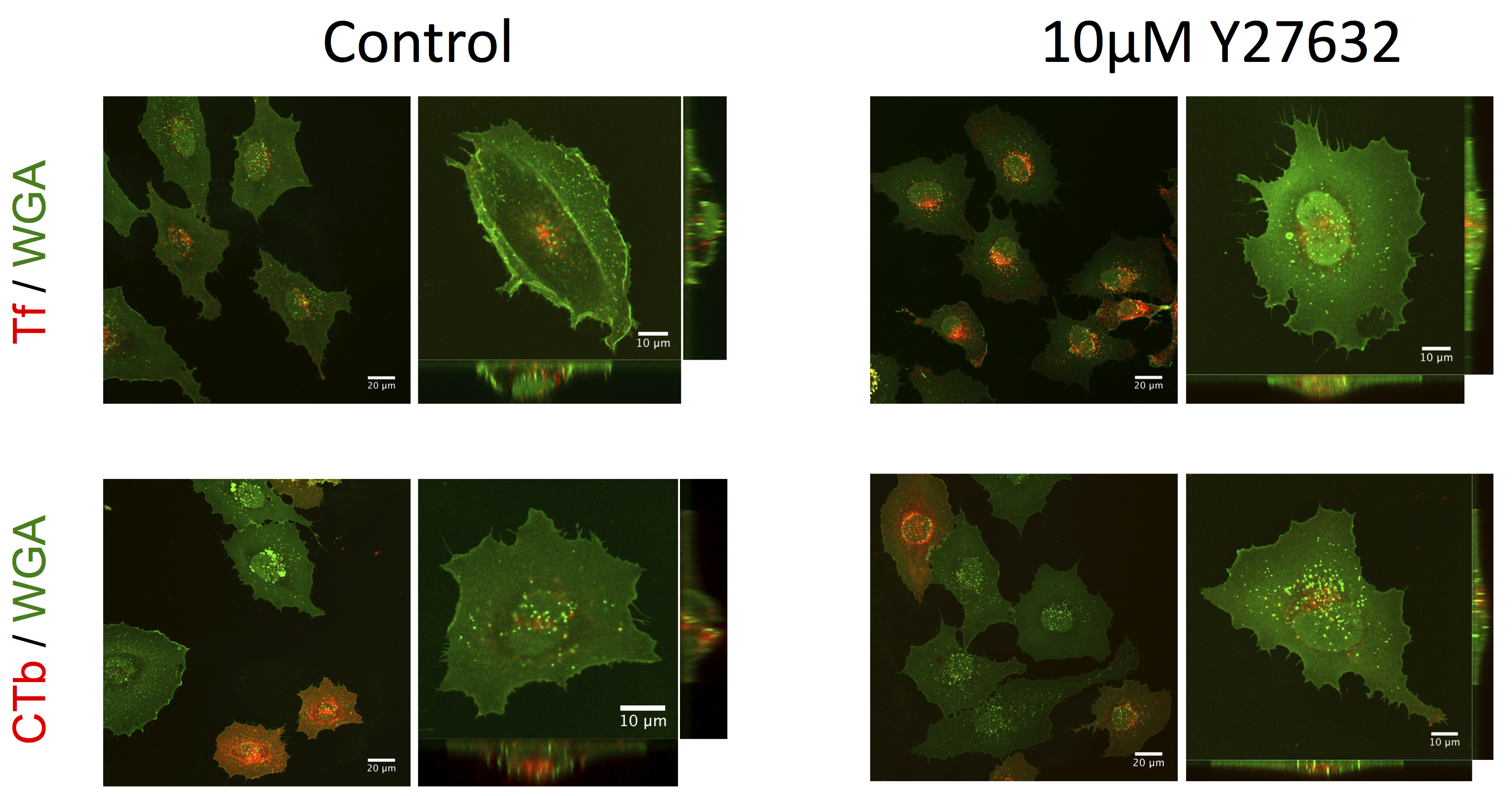

Supplement: Figure S5 — Rho kinase inhibition with Y27632 did not alter intracellular fluorescence pattern of Tf or CTb on REF52 cells. Confocal microscopy images of REF52 cells on FN-coated glass after 1-hour incubation with AF568-labeled markers, fixation and staining with WGA-AF488. Tf was internalized at similar numbers by cells and mainly localized at a perinuclear site, independently of Y27632 treatment (upper row), while CTb showed heterogeneous uptake efficiency among the cell population that was also independent of Y27632 treatment (lower row). (TIFF) [file pone.0096548.s005.tif]

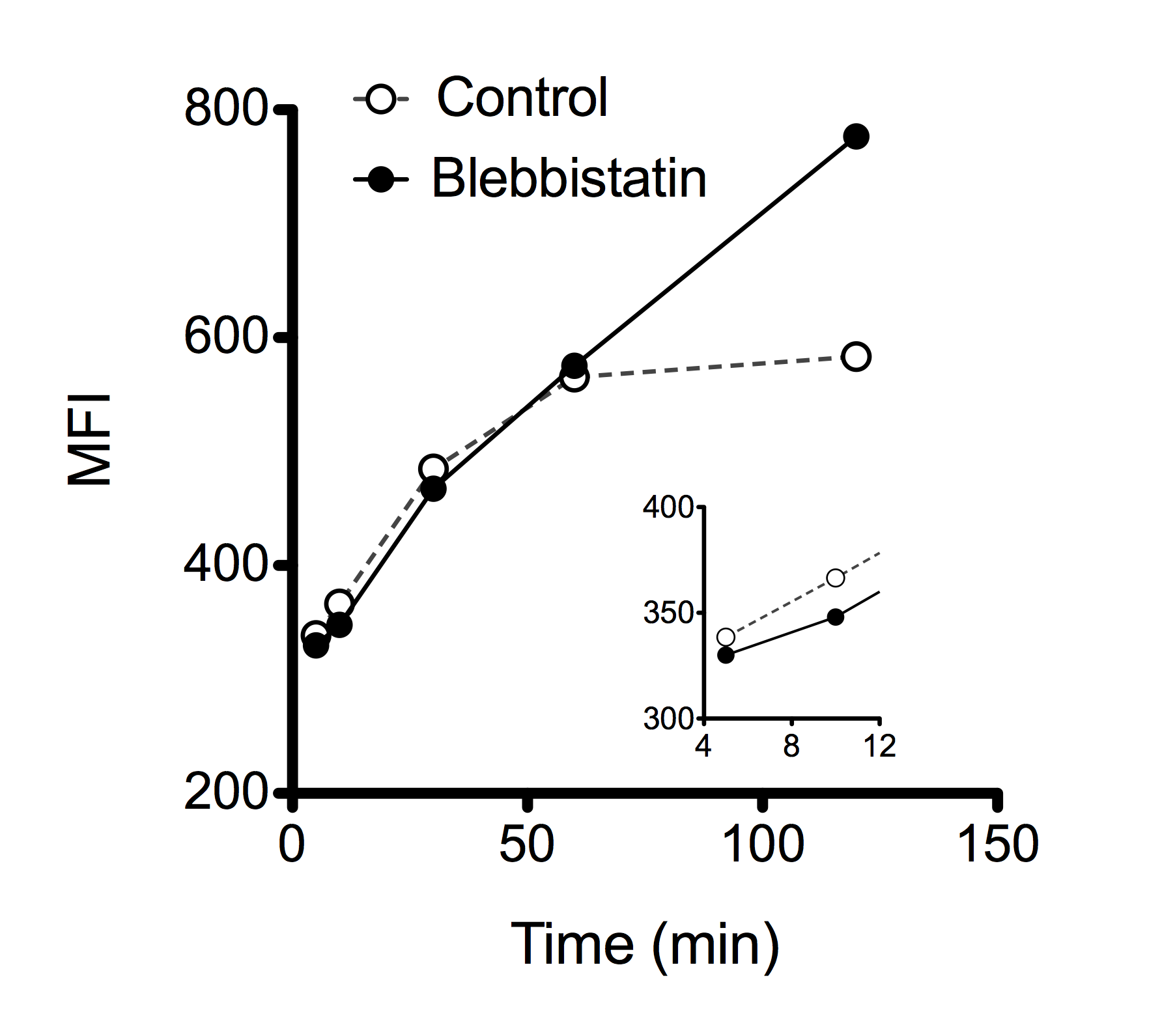

Supplement: Figure S6 — Blebbistatin treatment of REF52 cells affects Tf internalization and recycling kinetics. MFI of REF52 cells incubated with Tf at different time points on FN-coated plastic in the presence or absence of 50 µM blebbistatin. At short incubations blebbistatin inhibits Tf association by cells, while at longer time points the amount of Tf is enhanced compared to control conditions. The quasi-linear increase of MFI per cell indicates that blebbistatin has an effect of intracellular trafficking and recycling of Tf. Each data point represents the average of two experiments. (TIFF) [file pone.0096548.s006.tif]

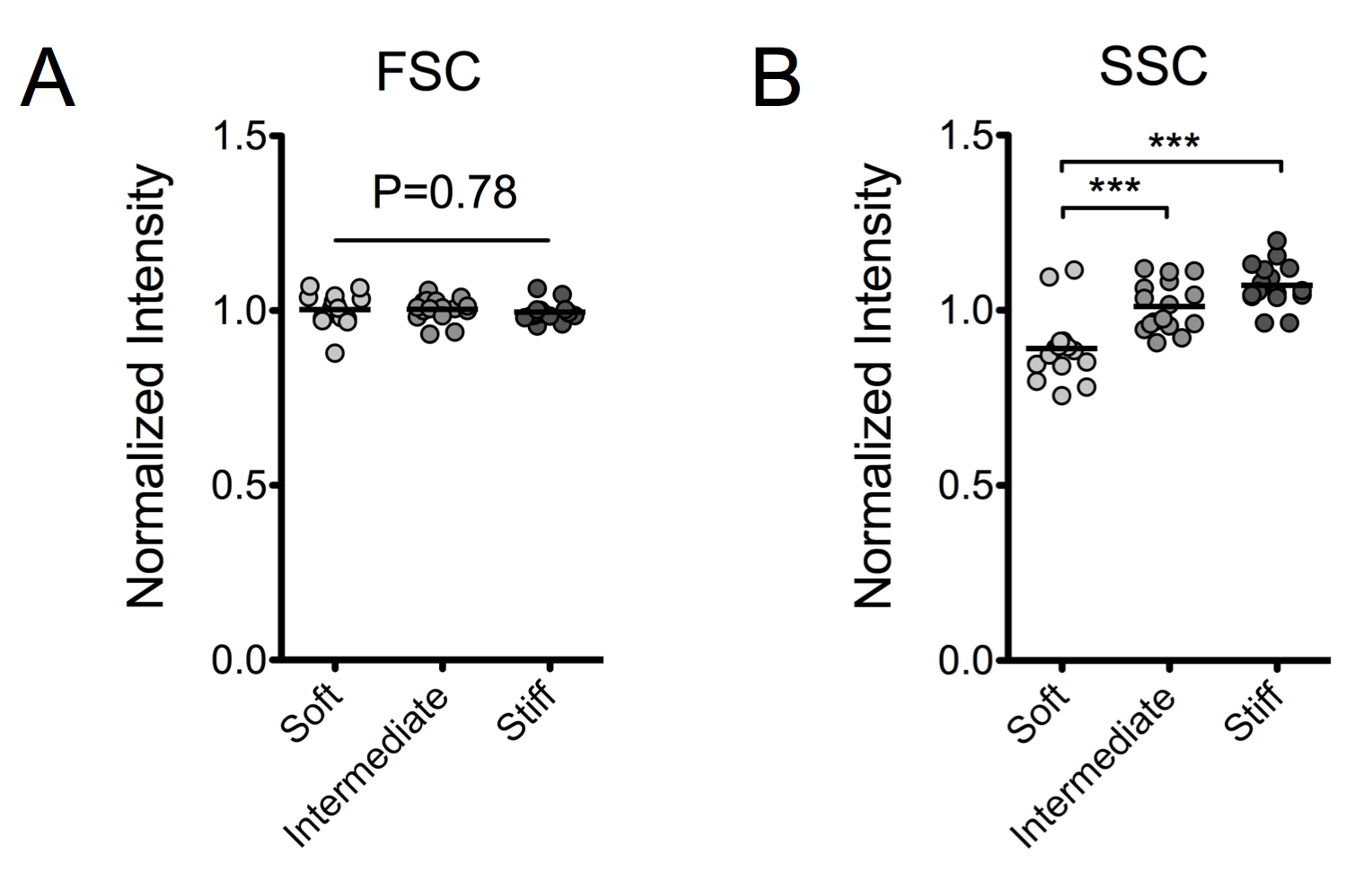

Supplement: Figure S7 — The SSC signal but not the FSC signal of REF52 cells depends on the elasticity of the substrate they were cultured on. Flow cytometry analysis of REF52 cells cultured on gels did not show a dependence of their FSC signal (A), while cells on soft gels showed a significantly lower SSC signal compared to cells cultured on intermediate or stiff hydrogels (B). Values from at least 4 independent experiments are presented with the value from each gel represented by a single dot and the mean value a solid line. (TIFF) [file pone.0096548.s007.tif]

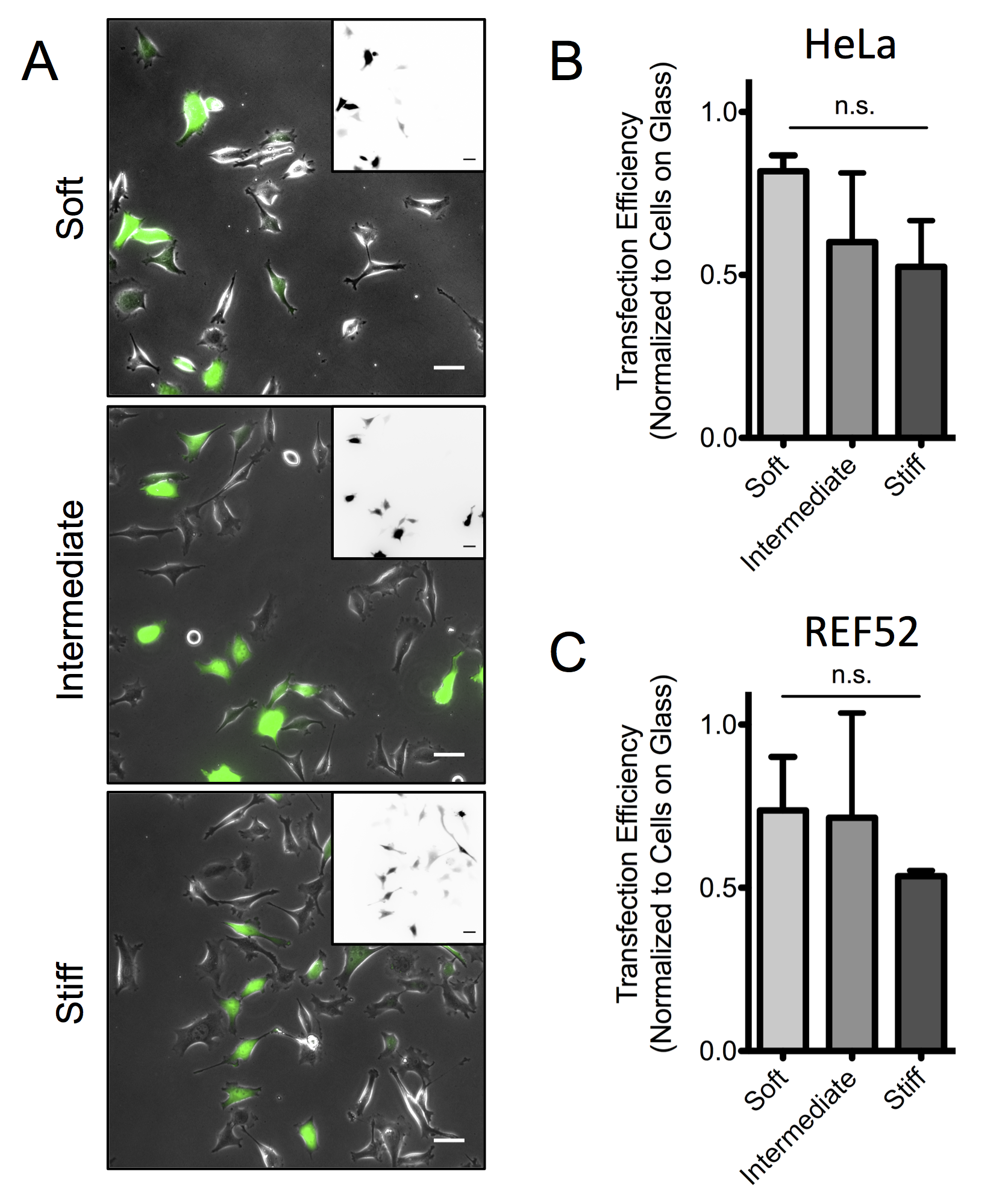

Supplement: Figure S8 — Transfection efficiency of HeLa or REF52 cells is not significantly affected by substrate elasticity. (A) Typical overlaid optical and fluorescence microscopy images of HeLa cells on FN-coated hydrogels of different elasticity used to calculate transfection efficiency. Insets show micrographs of the fluorescence channel (GFP). Scale bars: 50 µm. Transfection efficiency of (B) HeLa or (C) REF52 cells normalized to the value obtained on FN-coated glass. A non-significant decrease of the fraction of transfected cells with increasing stiffness was noted. Mean and SEM values from at least 3 gels and two independent experiments are presented (n = 200–1000 analyzed cells/gel). (TIFF) [file pone.0096548.s008.tif]

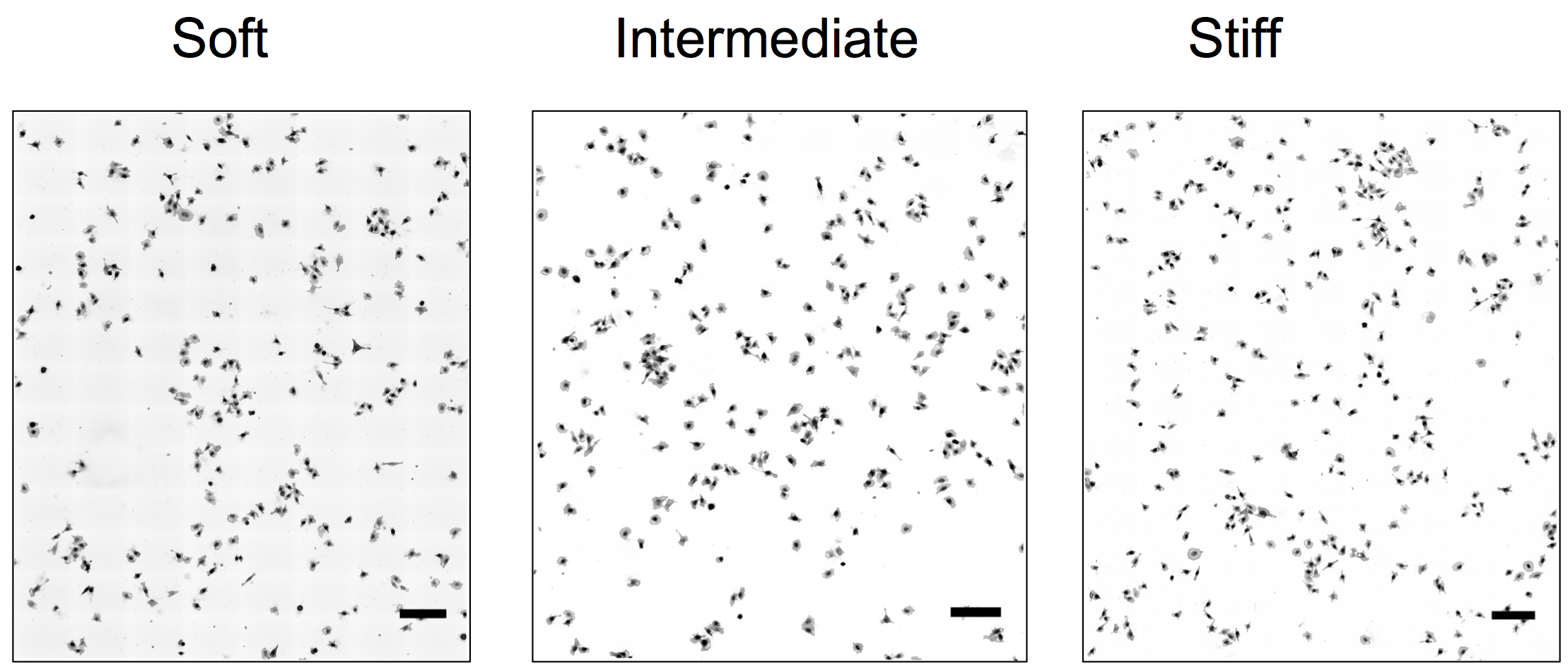

Supplement: Figure S9 — Cell density of HeLa cells does not depend on substrate elasticity. Inverted fluorescence microscopy images (stitched tiles) of HeLa cells on soft, intermediate and stiff hydrogels, stained with WGA. The amount of cells adhered was similar on all hydrogels, independent of their elasticity. Scale bars: 200 µm. (TIFF) [file pone.0096548.s009.tif]

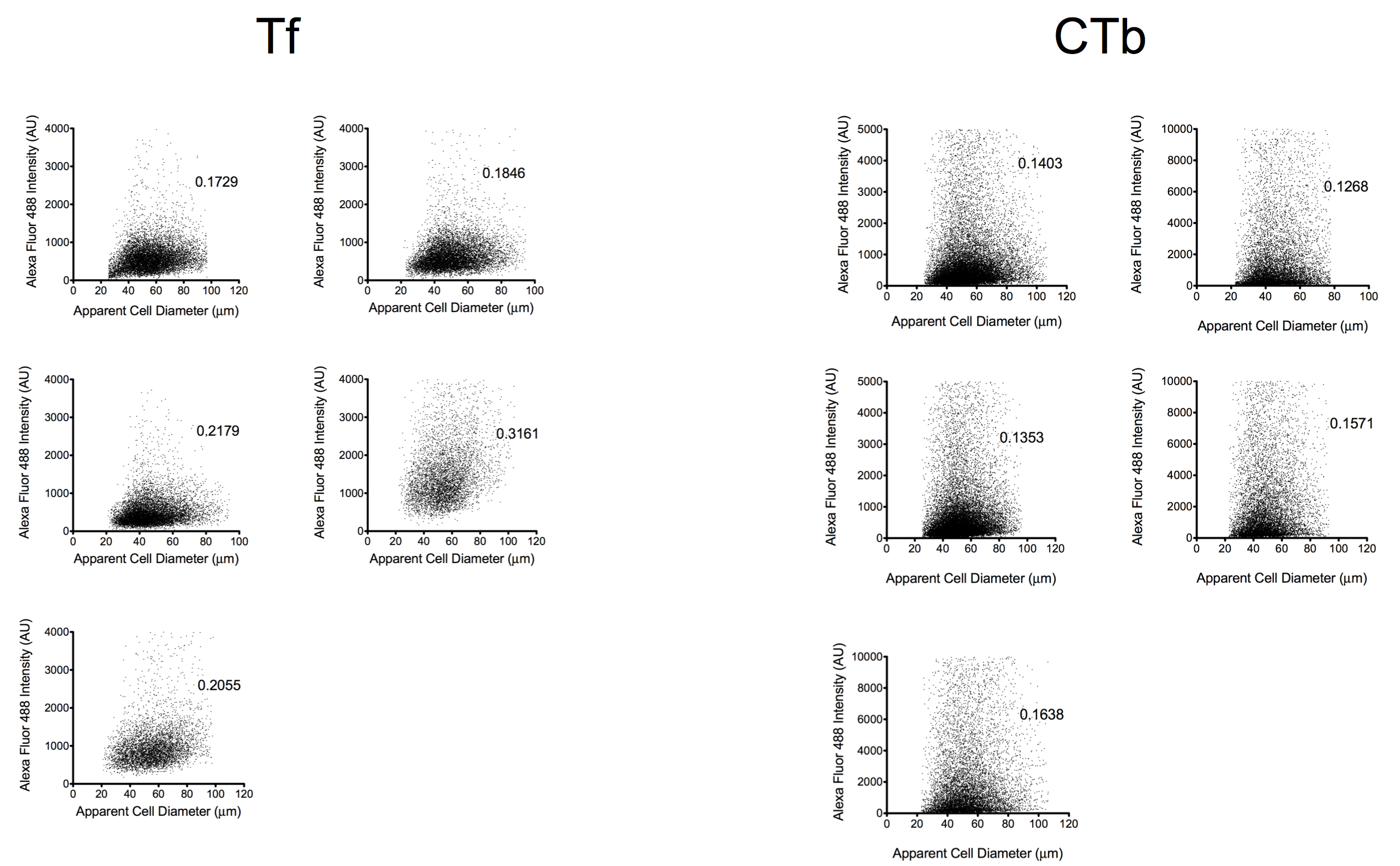

Supplement: Figure S10 — Tf or CTb association with cells is very weakly correlated to cell size. Dot plots of cell-associated fluorescence as a function of apparent cell diameter for Tf (left) or CTb (right) association. Data from 5 random experiments performed on cells on top of gels or glass are presented, along with the calculated Pearson's correlation coefficient. (TIFF) [file pone.0096548.s010.tif]
